# Maternal–prenatal stress and depression predict infant temperament during the COVID-19 pandemic

**DOI:** 10.1017/S0954579422001055

**Published:** 2022-11-08

**Authors:** Jessica L. Buthmann, Jonas G. Miller, Ian H. Gotlib

**Affiliations:** Department of Psychology, Stanford University, Stanford, CA, USA

**Keywords:** COVID-19, depression, infant temperament, prenatal mental health, stress

## Abstract

Researchers have begun to examine the psychological toll of the ongoing global COVID-19 pandemic. Data are now emerging indicating that there may be long-term adverse effects of the pandemic on new mothers and on children born during this period. In a longitudinal study of maternal mental health and child emotional development during the pandemic, we conducted online assessments of a cohort of women at two time points: when they were pregnant at the beginning of the surge of the pandemic in the United States (baseline, *N* = 725), and approximately 1 year postpartum (follow-up, *N* = 296), examining prenatal and postnatal maternal mental health, prenatal pandemic-related stress, and infant temperament. Pandemic-related stress at baseline was associated with concurrent depressive symptoms and infant negative affect at follow-up. Baseline maternal depressive symptoms were associated with follow-up depressive symptoms, which in turn were also associated with infant negative affect. Pandemic-related stress during pregnancy may have enduring effects on infant temperament. These findings have important implications for our understanding of the emotional development of children who were *in utero* during the COVID-19 pandemic.

Researchers have begun to examine the psychological toll of the ongoing global COVID-19 pandemic. Data are now emerging indicating that there may be long-term adverse effects of the pandemic on new mothers and on children born during this period. In a longitudinal study of maternal mental health and child emotional development during the pandemic, we conducted online assessments of a cohort of women at two time points: when they were pregnant at the beginning of the surge of the pandemic in the United States (baseline, *N* = 725), and approximately 1 year postpartum (follow-up, *N* = 296), examining prenatal and postnatal maternal mental health, prenatal pandemic-related stress, and infant temperament. Pandemic-related stress at baseline was associated with concurrent depressive symptoms and infant negative affect at follow-up. Baseline maternal depressive symptoms were associated with follow-up depressive symptoms, which in turn were also associated with infant negative affect. Pandemic-related stress during pregnancy may have enduring effects on infant temperament. These findings have important implications for our understanding of the emotional development of children who were *in utero* during the COVID-19 pandemic.

The COVID-19 pandemic has brought devastation on a global scale. More than six million people have died – more than 1,000,000 in the United States alone (WHO COVID-19 Dashboard, 2021). The economic turmoil, educational disruption, fear, bereavement, and isolation since the pandemic began have contributed to a marked increase in the prevalence of mental health difficulties. Researchers have now documented increases in symptoms of anxiety, depression, somatization, and PTSD within the first few months of the global outbreak of the pandemic, especially in women and in people under 40 years of age (Motrico et al., 2021; Ran et al., 2020; Xiong et al., 2020). It is critical that we continue to assess the psychological impact of this pandemic in order to elucidate and work to minimize its lasting deleterious effects, particularly in vulnerable populations.

Prior to the pandemic, investigators had tested the Developmental Origins of Health and Disease hypothesis (Gluckman et al., 2007), which posits that early experiences, including those occurring during the prenatal period, can lead to a cascade of alterations and adaptations with lifelong consequences for physical and mental health. Findings from these studies indicated that prenatal maternal stress and adversity, including natural disasters (Buthmann, Finik, et al., 2019; Nomura et al., 2019) and maternal difficulties with mental health (Acosta et al., 2019; Davis et al., 2020), are related to emotional difficulties in the offspring. It is important to recognize, however, that investigators have reported mixed findings concerning whether prenatal or postnatal maternal mental health symptoms are more strongly related to infant well-being (Erickson et al., 2017). Prenatal depression has been found to be a risk factor for postnatal depression (Edwards et al., 2008). In fact, the two variables are often collinear (van der Wal et al., 2007) and prenatal depressive symptoms have been found to not predict offspring well-being after controlling for postnatal depressive symptoms (Buthmann, Ham, et al. 2019; Rouse & Goodman, 2014).

Researchers have now begun to examine prenatal maternal mental health during the pandemic, as well as factors that predict adverse outcomes in mothers and their children. Most studies in this area have assessed maternal symptoms of depression during the prenatal period. For example, King et al. (2021) found that a sample of women who were pregnant at the beginning of the U.S. surge of the COVID-19 pandemic had higher levels of depressive symptoms than did a matched sample of women who were pregnant several years prior to the pandemic. King et al. found further that symptoms of depression during the pandemic were higher in women who reported greater fear of infection, financial difficulty, being a person of color, and changes to their prenatal care. Other researchers have also reported increases in depressive symptoms during the pandemic in pregnant women who experienced a lack of social support (Filippetti et al., 2022), financial instability (Sherin et al., 2021), and a reduced ability to exercise regularly (Gildner et al., 2020).

In addition to depressive symptoms, investigators have also documented elevated levels of pandemic-related stress and anxiety in pregnant women. Symptoms of anxiety have been found to be related to greater fear of infection (Colli et al., 2021) and to changes in prenatal care or in birth plans (Groulx et al., 2021). Feeling unprepared for labor and delivery was found to be associated with medical vulnerability (e.g., chronic illness or having a high-risk pregnancy), changes to prenatal care, and not having access to outdoor space (Preis et al., 2020). In a cross-sectional sample of pregnant women and new mothers, Nomura et al. (2021) found that concurrent suicidal ideation and substance use was highest in women who tested positive for the virus, and that levels of distress were highest for women in the third trimester and postpartum periods. Fear of the baby contracting COVID-19, lack of social support, and higher levels of pregnancy-specific anxiety have been found to be associated with poorer overall mental health (Pope et al., 2021). In China, Xie et al. (2021) found that symptoms of somatization, depression, anxiety, and hostility in women who were pregnant in 2020 were higher than in women who were pregnant in 2019, prior to the pandemic. Further, pregnant healthcare workers in China were found to have higher levels of symptoms of somatization, anxiety, and hostility than nonpregnant female healthcare workers (Liu & Pérez-Edgar, 2019). It is important to note, however, that this literature has focused on the concurrent associations between pandemic-related stress and prenatal maternal mental health; it is unclear whether pandemic-related stress is prospectively associated with postnatal maternal mental health.

In addition to the psychological toll exacted by symptoms of depression and stress, further concern about the rise in mental health difficulties in new and expecting mothers during the pandemic stems from the association between these difficulties and infant development. For example, symptoms of maternal depression during the prenatal period have been related to increased negative affect in infants, including lack of smiling, difficulty soothing, and increased sadness (Davis et al., 2007; Huynh et al., 2014). Other researchers, however, have found that postnatal, but not prenatal, depressive symptoms predict offspring negative affect (Buthmann, Ham, et al., 2019; Rouse & Goodman, 2014). Further, prenatal maternal stress has been linked consistently with infant negative emotionality. Stress related to natural disasters (Buthmann, Ham, et al., 2019; Laplante et al., 2016), intimate partner violence (Gibson et al., 2015), pregnancy-specific anxiety (Blair et al., 2011), and stressful life events (Robinson et al., 2011) have been associated with increased negative affect in infant and toddler offspring. Infant negative affect, in turn, has been shown to predict the development of psychology symptoms later in life (De Pauw & Mervielde, 2010; Lahey et al., 2008; Rettew & McKee, 2005).

To date, few researchers have assessed the effects of maternal prenatal mental health difficulties on infant functioning during the pandemic. In Italy, Provenzi, Grumi, et al. (2021) reported that high levels of prenatal emotional stress and low levels of social support during the pandemic were related to higher symptoms of prenatal maternal anxiety, and that maternal parenting stress and bond with the child mediated the association between prenatal maternal anxiety and reduced regulatory capacity in 3-month-old infants. This research group also found that prenatal maternal stress related to the pandemic was associated with increased *SLC6A4* methylation in neonatal buccal samples, which in turn predicted decreased surgency/positive affect in infants at 3 months of age (Provenzi, Mambretti, et al., 2021). Another research group found that infants who were *in utero* during the pandemic scored lower on gross and fine motor function and personal–social function than did infants who were *in utero* prior to the pandemic, regardless of maternal COVID-19 infection (Shuffrey et al., 2022) and, further, that infant temperament was related to maternal pandemic-related stress, not to maternal infection (Bianco et al., 2022). In contrast, Fiske et al. (2021) found that although postnatal maternal symptoms of depression increased over the course of the pandemic, there was no relation between this increase in symptoms and difficulties in infant temperament. Taken together, researchers have reported mixed findings regarding whether the effects of pandemic-related stress on infant temperament are mediated by, or are independent of, maternal difficulties in mental health.

The rise in mental health difficulties in relation to the COVID-19 pandemic underscores the need to examine links between maternal prenatal mental health and subsequent postnatal maternal and infant functioning. To examine these associations, we collected data from women who were pregnant at the beginning of the surge of COVID-19 in the United States and followed up with these women at approximately 1 year postpartum. We hypothesized that prenatal maternal pandemic-related stress and prenatal depressive symptoms will be positively associated with postnatal depressive symptoms (Hypothesis 1). We also expected that prenatal pandemic-related stress and postnatal maternal depressive symptoms will be associated with infant temperament, specifically with elevated infant negative affect (Hypothesis 2). Given the mixed findings in the literature regarding the relation between prenatal maternal depressive symptoms and offspring temperament, we examined this association but did not generate a specific prediction.

## Methods

### Participants

**Figure 1.**
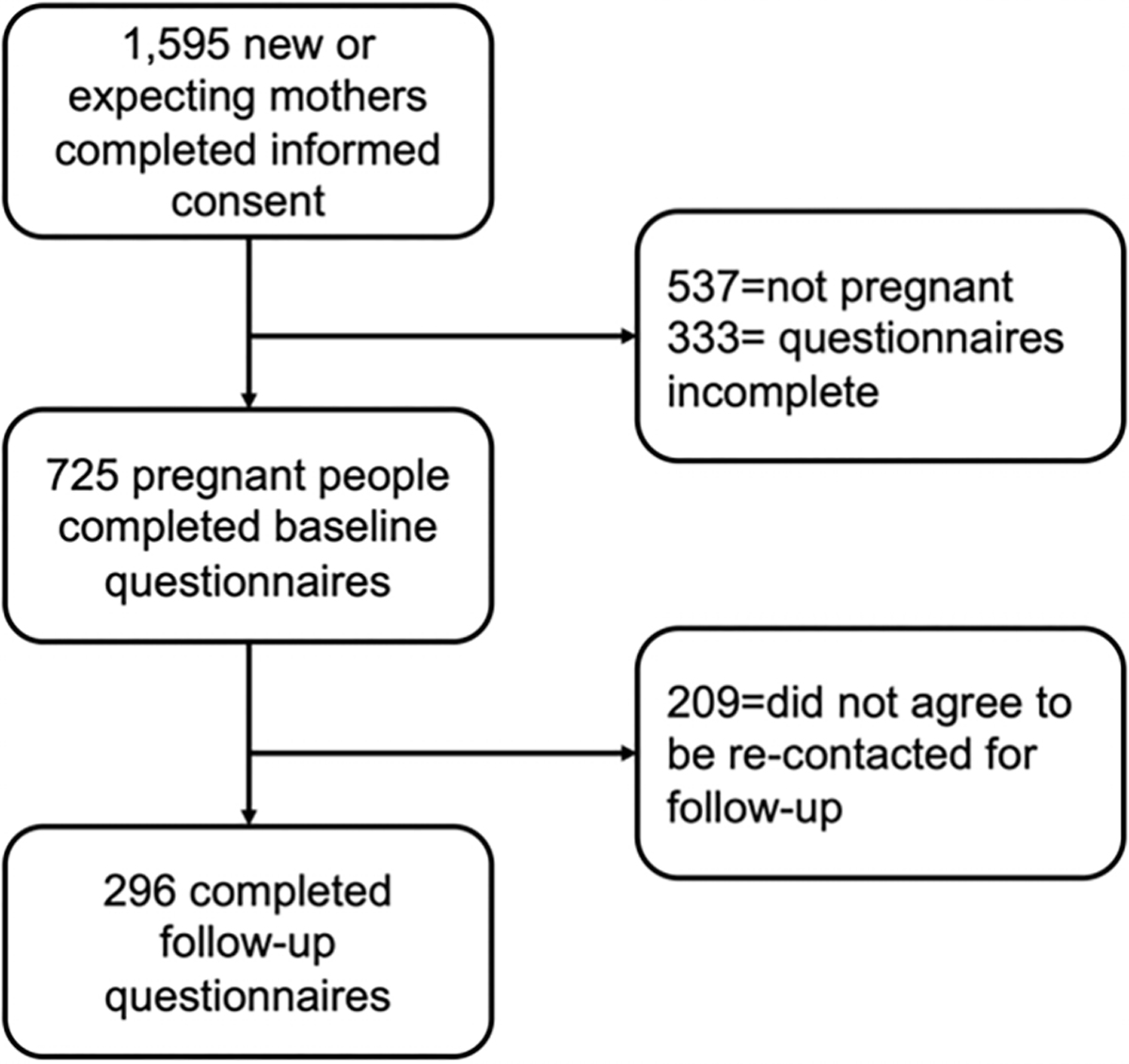
Consort flow diagram of study recruitment, attrition, and completion.

We recruited women to participate in the [Stanford COVID-19 Perinatal Experiences] COVID-19 Perinatal Experiences (COPE) project via online advertisements in April–May of 2020 (baseline), as the COVID-19 pandemic surged in the United States (see King et al., 2021 for the full study description). Advertisements were placed online, on social media sites including Facebook and Twitter, soliciting participation in a study of women during the COVID-19 outbreak. Advertisement audience criteria included ages 18–45, within 50 miles of [blinded for review], and interests in “prenatal care,” “motherhood,” and “what to expect when you’re expecting.” Criteria for inclusion in the broader study were that participants be either pregnant or less than 6 months postpartum, and at least 18 years of age. One thousand five hundred and ninety five women provided informed consent, of whom 1,058 were pregnant (see Figure 1 for consort diagram). Seven hundred and twenty five pregnant women completed the survey in full. Five hundred and sixteen of these women provided contact information and consented to be contacted in the future for follow-up. We followed up at approximately 1 year postpartum in August–September 2021 (follow-up) and received responses from 296 participants.

### Measures

#### Demographic characteristics

At baseline, participants reported their age, race, educational attainment, household size, and 2019 household income. At follow-up, participants reported the date of birth of the child with whom they were pregnant at baseline. We calculated an income-to-needs ratio based on household size and income using the 2019 federal poverty level per household size. See Table 1 for demographic variables of the samples that completed assessments at baseline and follow-up and baseline only.

#### Pandemic-related stress

At baseline we administered the COVID-19 and Perinatal Experiences Impact Survey (COPE-IS; Thomason et al., 2020). This survey includes questions about COVID-19 symptoms and test results, COVID-19-specific distress related to the birth (e.g., “*Are you concerned about possible future changes in support and involvement of your family and friends in your baby’s birth as a result of the COVID-19 outbreak?”, “How concerned are you [about your child’s health as a result of the COVID-19 outbreak]?*”), related to disrupted social support, about current and future expected financial impact, and overall impact. Distress is rated on a Likert scale from not distressing (0) to highly distressing (7). The survey also includes questions about general health, prenatal experiences (e.g., changes to medical care, pregnancy complications), and postnatal experiences for participants who have already given birth (e.g., changes to birthing plan or postnatal care).

#### Maternal depressive symptoms

We administered the Edinburgh Postnatal Depression Scale (EPDS; Cox et al., 1987) at baseline and at follow-up. The EPDS is validated for use during and after pregnancy (Bergink et al., 2011). This 10-item self-report scale asks participants to rate the degree to which statements accurately described their mood in the past 7 days. Participants respond on a scale of 0 (never) to 3 (most of the time), yielding a possible range of 0–30.).

#### Infant temperament

At follow-up, participants reported on their child’s temperament using the Infant Behavior Questionnaire – Very Short Form (IBQ-VSF; Putnam et al., 2014). This 37-item measure asks mothers to indicate the frequency with which their child engaged in specific behaviors in the past 7 days on a scale from 1 (never) to 7 (always). Items are averaged to form three subscales: negative affect (e.g., *When tired, how often did your baby show distress), surgency/positive affect (e.g., How often during the week did your baby move quickly toward new objects), and effortful control (e.g., How often during the last week did the baby play with one toy or object for 5–10 min?*).

### Analysis

#### Prenatal COVID-19 stress

We computed descriptive statistics and correlations among variables of interest using IBM SPSS 28 (Armonk, NY). We entered the following 15 z-scored indicators of prenatal pandemic-related stressors from the COPE-IS into a PCA with direct oblimin rotation: changes to prenatal care, current financial impact, expected future financial impact, concern about changes to medical birthing team, concern about social support during birth, concern about caring for the child after birth, concern about the child’s health, concern about own health, concern about family’s health, concern about reduced access to resources, impact of the pandemic on daily life, valence of the impact of the pandemic on daily life, distress about disrupted social support, somatization symptoms, and rating of current social support. Changes to prenatal care, current social support, and somatization symptoms were removed because their factor loadings were below .500. Bartlett’s test of sphericity was significant, *X*^2^(66) = 713.55, *p* < .001, and the Kaiser–Meyer–Olkin measure of sampling adequacy was .78, indicating these data were suitable for PCA.

#### Path analysis

Scores for prenatal pandemic-related stress from the PCA, prenatal depressive symptoms, postnatal depressive symptoms, income-to-needs ratio, and child sex and age at follow-up were entered into a path analysis predicting infant negative affect using the lavaan package for RStudio (Rosseel, 2012). FIML estimation was used to account for missing data. Little’s test indicated that data were missing completely at random at baseline (*X*^2^(11) = 12.42, *p* = .33) and at follow-up (*X*^2^(18) = 12.01, *p* = .89). Thus, our data appear to meet the missingness assumptions of FIML. See supplemental tables S1 and S2 for the frequency of missing data at baseline and follow-up.

We examined the associations between prenatal maternal pandemic-related stress and prenatal depressive symptoms predicting postnatal depressive symptoms, and between prenatal pandemic-related stress and postnatal depressive symptoms predicting infant negative affect. We used nonsignificant *X*^2^ values, CFI > .95, RMSEA < .06, and SRMR < .05 as indicators of good model fit (Hu & Bentler, 1999; Schumacker & Lomax, 2012). We adjusted for multiple comparisons using a false discovery rate procedure (Benjamini & Hochberg, 1995).

#### Prenatal and postnatal depressive symptoms

To examine the relation between prenatal versus postnatal depressive symptoms and infant negative affect, we used a clinical cutoff score of 12 to categorize participants as below or above this score in the prenatal or postnatal period. We then conducted an ANOVA with the binary prenatal and the binary postnatal depression scores predicting infant negative affect. We used Bonferroni correction for multiple comparisons.

## Results

### Descriptive statistics

Demographic characteristics are presented in Table 1. The sample was majority White with a relatively high income-to-needs ratio compared to the federal poverty level. Compared to the those who only participated at baseline, those who participated at baseline and in the follow-up assessment were less likely to be Hispanic/Latinx (*X*^2^(1) = 7.46, *p* = .006) and more likely to be White (*X*^2^(1) = 13.99, *p* < .001), more likely to have a graduate or professional degree (*X*^2^(1) = 6.06, *p* = .014), to have higher income ((722) = −2.58, *p* = .005), and to be older (*t*(627.9) = −2.84, *p* = .002). Means and SDs of relevant study variables are presented in Table 2. The average age of the offspring at follow-up was approximately 13 months. Depressive symptoms were significantly higher at baseline (*M* = 9.38, *SD* = 4.96) than at follow-up (*M* = 7.65, *SD* = 5.06, *F*(1) = 41.55, *p* < .001).

### Prenatal COVID-19 stress

Three components with an eigenvalue above 1.0 were identified by the PCA (see Table 3 for component loadings). The first component, accounting for 30.7% of the variance, included general concerns about the pandemic, such as impact on daily life and changes to medical birthing team. The second component, accounting for 10.95% of the variance, was composed of two item assessing concerns about current and future financial impact. The third, accounting for 10.09% of the variance, was composed of two item assessing concerns about the health of the participant and the participant’s family. Given that most of the items loaded on the first component, which had the largest eigenvalue, we focused on this component in the analyses that follow.

Correlations among the study variables are shown in Table 2. The prenatal pandemic-related stress component was positively correlated with prenatal and postnatal depressive symptoms and with infant negative affect. Postnatal, but not prenatal, depressive symptoms were positively correlated with infant negative affect. Scatterplots illustrating these associations are presented in Supplementary Figure 1a–d.

### Path analysis

**Figure 2.**
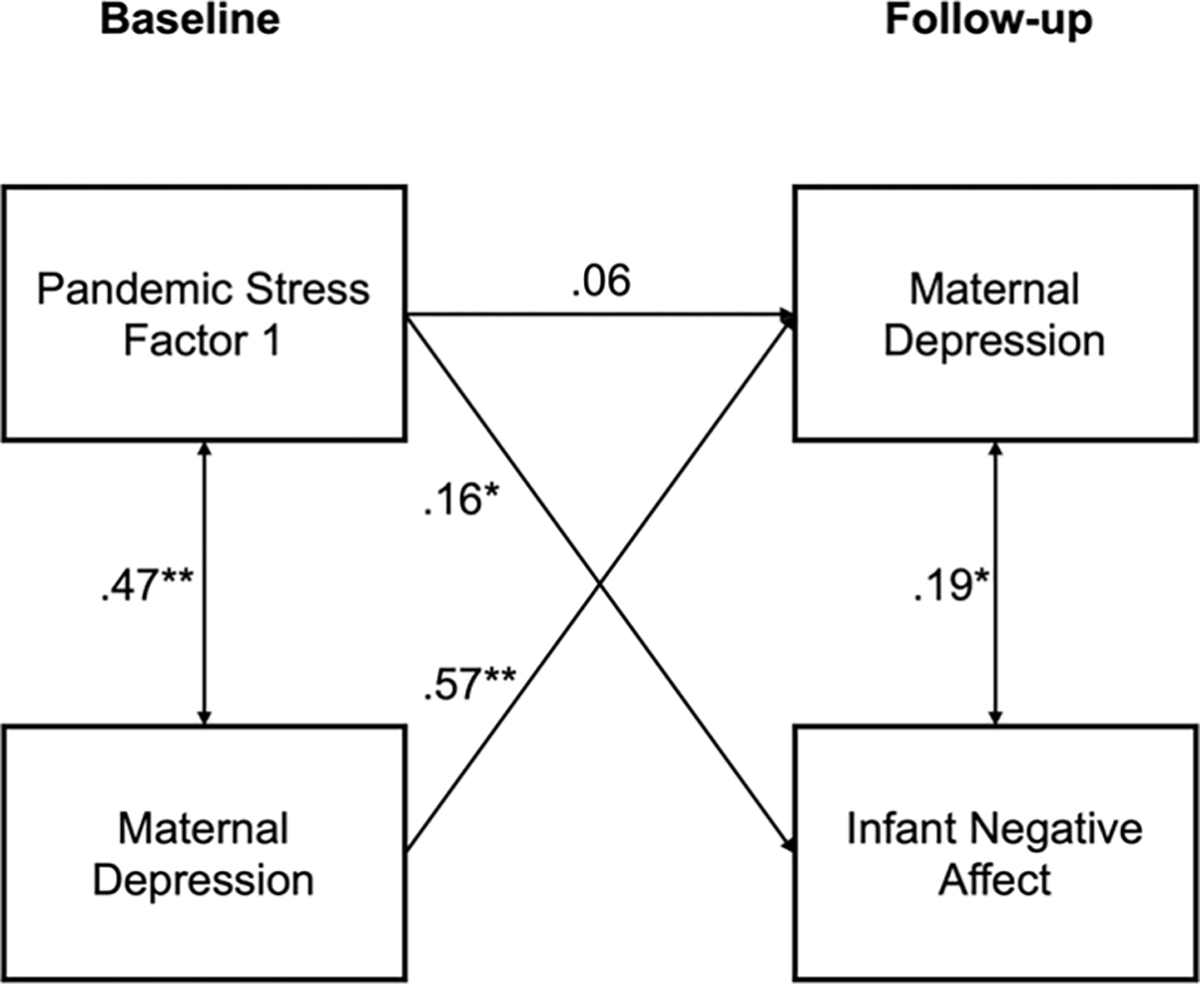
Path diagram of prenatal pandemic stress and postnatal depressive symptoms predicting infant negative affect. Values represent standardized path coefficients. **p < .001, *p < .05.

In a path analysis we modeled paths in which postnatal depressive symptoms were regressed on prenatal pandemic-related stress, prenatal depressive symptoms, and income-to-needs ratio; infant negative affect was regressed on prenatal pandemic-related stress, income-to-needs ratio, child sex and child age at follow-up, and covaried with postnatal depressive symptoms. Similar to the findings from the correlation analysis, the path from prenatal depressive symptoms to infant negative affect was not statistically significant (*β*= −0.04, *p* = .58). Therefore, to increase model parsimony, we removed this path from the final model, which is visually depicted in Figure 2. Four model fit indicators suggested good fit to the data (CFI = .97, SRMR = .04, and RMSEA = .05, *p* = .47; *X*^2^(9) = 15.78, *p* = .07). Path coefficients are presented in Table 4.

We hypothesized that prenatal maternal pandemic-related stress and prenatal depressive symptoms will be positively associated with postnatal depressive symptoms (Hypothesis 1). As expected, prenatal depressive symptoms were associated with postnatal depressive symptoms (standardized *β* = .57, unadjusted *p* < .001, *q* < .001). Pandemic-related stress was also positively associated with postnatal depressive symptoms, although this effect was not statistically significant after covarying for prenatal symptoms and income-to-needs ratio and adjusting for multiple comparisons (*β* = .06, unadjusted *p* = .19, *q* = .34). Income-to-needs ratio was not significantly associated with postnatal depressive symptoms (*β* = −.06, unadjusted *p* = .26, *q* = .41). Finally, prenatal depressive symptoms were correlated significantly with prenatal pandemic-related stress (*r* = .47, unadjusted *p* < .001, *q* < .001).

We also predicted that prenatal pandemic-related stress and postnatal maternal depressive symptoms will be positively associated with infant negative affect (Hypothesis 2). As expected, pandemic-related stress (β = .19, unadjusted *p* = .001, *q* = .003) was associated with infant negative affect. Income-to-needs ratio was also associated with infant negative affect (*β* = −.19, unadjusted *p* = .001, *q* = .004); child sex and age at T2 were not associated significantly with infant negative affect. Postnatal maternal depressive symptoms (*r* = .19, unadjusted *p* = .001, *q* = .003) were significantly correlated with infant negative affect.

### Clinical depressive symptom cutoffs

**Figure 3.**
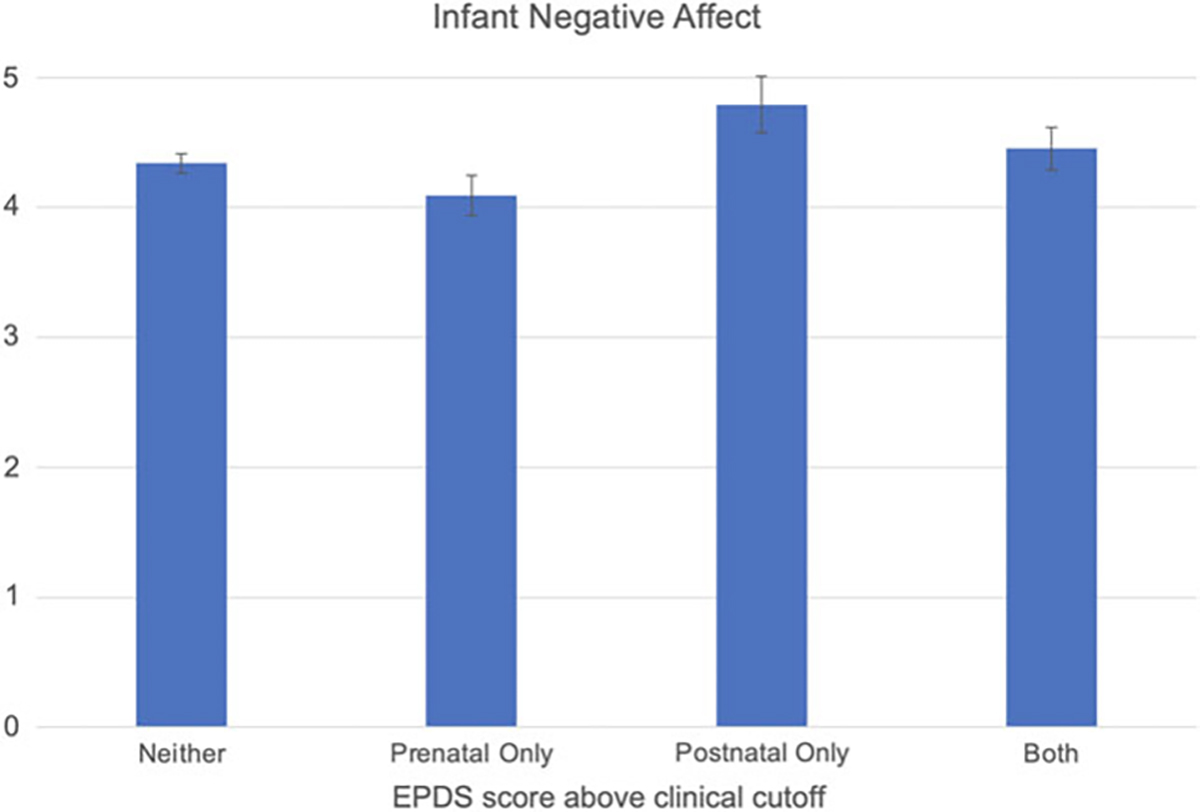
Infant negative affect by prenatal and postnatal maternal depressive symptoms. Presence of depression determined using a clinical cutoff score of 12 on the Edinburgh Postnatal Depression Scale (EPDS).

We created two dichotomous variables using a clinical cutoff score of 12 on the EPDS to identify groups of individuals with and without significant depressive symptomatology at the prenatal and postnatal assessments. Two thirds (65.6%) of the participants did not have depressive symptoms above the cutoff at either time point; 14.3% had high levels of symptoms only in the prenatal period; 7.3% had high levels of symptoms only in the postnatal period; and 12.8% of the participants had high levels of symptoms at both the prenatal and postnatal assessments. A two-way (prenatal depression [low, high] by postnatal depression [low, high]) ANOVA with infant negative affect as the dependent variable yielded only a main effect for postnatal depression (*F*(1, 269) = 6.36, *p* = .012, partial *η*^2^ = .023); mothers with high levels of depressive symptoms in the postnatal period reported higher infant negative affect (*M* = 4.62, *SD* = 0.14) than did mothers with low levels of postnatal depressive symptoms (*M* = 4.22, *SD* = 0.09; see Figure 3). Neither the main effect of prenatal depression (*F*(1, 269) = 3.31, *p* = .070, partial *η*^2^ = .012) nor the interaction of prenatal and postnatal depression (*F*(1, 269) = 0.07, *p* = .790, partial *η*^2^ = .000) was significant.

## Discussion

In this study we examined the relations among prenatal maternal stress related to the COVID-19 pandemic, prenatal and postnatal maternal depressive symptoms, and infant negative affect. We found that prenatal pandemic-related stress was associated with more severe prenatal maternal depressive symptoms, as well as with infant negative affect; postnatal, but not prenatal, depressive symptoms were also significantly related to infant negative affect.

We expected that prenatal pandemic-stress would be positively associated with both prenatal and postnatal depressive symptoms. Although the zero-order correlations among the variables were positive and significant, the path analysis indicated that the association between prenatal pandemic stress and postnatal depressive symptoms was not statistically significant after controlling for prenatal depressive symptoms and income-to-needs ratio. In addition, the effect of prenatal depressive symptoms on postnatal depressive symptoms was much larger than the effect of prenatal pandemic-related stress on postnatal depressive symptoms. Taken together, our findings indicate that prenatal pandemic-related stress is associated with elevated concurrent depressive symptoms, which in turn predict postnatal depressive symptoms. The association between prenatal pandemic-related stress and concurrent depressive symptoms may indeed be reciprocal. As reviewed by McLaughlin et al. (2022), people with higher levels of psychopathology symptoms, including depressive symptoms, are less likely to engage in cognitive reappraisal of stressful events or use other beneficial coping strategies. In the context of the COVID-19 pandemic, individuals’ inability to cope successfully with stressors may exacerbate their depressive symptoms, which, in turn, may heighten their subjective stress experience of the pandemic. More research is needed to elucidate the relation between pandemic-related stress and depressive symptoms over the longer term, especially in new mothers.

We found that depressive symptoms were higher at baseline, during the initial stages of the pandemic, than they were at follow-up, approximately 16 months later. Consistent with our findings, several other studies have reported that symptoms of psychological distress that surged with the onset of the pandemic have now decreased. For example, López-Morales et al. (2021) found that prenatal maternal symptoms of depression and anxiety increased early in the pandemic following stay-at-home orders in Argentina and decreased slightly over time. Longitudinal studies of mental health symptoms in nonpregnant people have also shown a decrease in symptoms since the beginning of the pandemic (Fluharty et al., 2021; Yarrington et al., 2021).

Our results are largely consistent with findings reported prior to the pandemic of associations between poor prenatal maternal mental health, whether defined as stress (e.g., Buthmann, Finik, et al., 2019), depression (e.g., Borchers et al., 2021), or anxiety (e.g., Van den Bergh et al., 2008), and subsequent difficulties in offsprings’ emotional development. We found that prenatal levels of pandemic-related stress, but not prenatal maternal depressive symptoms, predicted subsequent infant negative affect. Prior work has similarly found that prenatal maternal depressive symptoms do not predict infant temperament after controlling for postnatal depressive symptoms (Buthmann, Finik, et al., 2019; Davis et al., 2007; Rouse & Goodman, 2014). Although we did not collect biological specimens in our study to elucidate mechanisms that might underlie the relations between maternal mental health and infant temperament, research testing the Developmental Origins of Health and Disease hypothesis (Gluckman et al., 2007) has implicated epigenetic, neuroendocrine, and/or inflammatory pathways in this association. Future research should examine whether the elevated levels of negative affect found in infants of mothers who experienced high stress during the pandemic will be sustained or normalize over time, and whether levels of negative emotionality in these infants predict the subsequent development of psychopathology, as has been documented by other investigators (Abulizi et al., 2017; Liu & Pérez-Edgar, 2019).

We should note four limitations of this study. First, our sample was composed primarily of White families of high socioeconomic status. There was also participant attrition between the first and second assessments; white and college educated women were more likely to participate at both timepoints. The relatively high proportion of white and affluent participants may be due to our recruitment through the internet and social media sites. Therefore, we should be cautious about the generalizability of our findings to other populations. Second, data were self-reported via an online survey, and it is possible that participants were biased in their responses. This is particularly important for the reporting of infant negative affect, given that maternal depressive symptoms may have increased mothers’ sensitivity to infant negative affect; we should note, however, that recent studies have found little evidence of systematic reporting bias as a function of parental psychopathology (e.g., Olino et al., 2020). Third, although we demonstrated that maternal prenatal pandemic-related stress was associated with infant negative affect, we did not assess the same pandemic-related stressors in the postnatal period and, therefore, cannot determine the specificity of stress effects on infant negative affect to the prenatal period. Finally, given the low rate of positive COVID-19 infections in this sample, we cannot examine associations among infection, maternal mental health, and infant affect. However, recent research indicates that, regardless of maternal COVID-19 infection, infants who were *in utero* during the pandemic had poorer developmental outcomes than did infants who were *in utero* prior to the pandemic (Bianco et al., 2022; Shuffrey et al., 2022).

Despite these limitations, we document in this study that maternal prenatal pandemic-related stress at the beginning of the COVID-19 surge in the United States is associated with elevated concurrent maternal symptoms of depression and with infant negative affect. Further research is needed to examine trajectories of mental health symptoms of mothers who gave birth during the pandemic and their associations with trajectories of offspring emotional development. Elucidating factors that affect risk and resilience in children *in utero* during the COVID-19 pandemic will help public health officials and healthcare providers to identify groups in need of more resources or support in order to mitigate the psychological toll of the pandemic.

## Supplementary Material

1

## Figures and Tables

**Table 1.** Demographic characteristics

	Baseline and follow-up	Baseline only	Group comparison
	*M* (*SD*)	*M* (*SD*)	

Maternal age	34.27 (4.04)	33.31 (4.55)	*t*(627.9) = −2.84, *p* = .002*

Income-to-needs ratio	8.98 (4.01)	8.17 (4.18)	*t*(722) = −2.58, *p* = .005

Weeks pregnant at baseline	25.80 (8.76)	27.39 (8.80)	*t*(723) = 2.35, *p* = .009

Child age at T2	13.37 (2.18)	-	-

Maternal race			

Black	0.7%	1.8%	*X*^2^(1) = 1.35, *p* = .246

Native American	1.1%	0.9%	*X*^2^(1) = 0.08, *p* = .775

Native Hawaiian	1.8%	1.1%	*X*^2^(1) = 0.66, *p* = .417

Asian	16.8%	19.2%	*X*^2^(1) = 0.65, *p* = .419

Hispanic	9.9%	17.3%	*X*^2^(1) = 7.46, *p* = .006

White	75.8%	62.4%	*X*^2^(1) = 13.99, *p* < .001

Other	2.9%	2.0%	*X*^2^(1) = 0.66, *p* = .418

Maternal education			

Partial high school	0.4%	1.1%	*X*^2^(1) = 1.14, *p* = .287

High school/GED	1.1%	2.7%	*X*^2^(1) = 2.03, *p* = .154

Partial college	4.8%	7.5%	*X*^2^(1) = 2.14, *p* = .144

Associates degree	3.7%	5.3%	*X*^2^(1) = 1.03, *p* = .310

Trade school/apprenticeship	0.4%	2.0%	*X*^2^(1) = 3.30, *p* = .069

Bachelor’s degree	33.0%	33.6%	*X*^2^(1) = 0.03, *p* = .855

Graduate school	56.8%	47.3%	*X*^2^(1) = 6.06, *p* = .014

Child sex (% female)	51.7%	-	-

Note:

* denotes equal variances not assumed.

**Table 2.** Correlations among study variables

	1	2	3	4	5	6
1. Income-to-needs ratio	-					
2. Child age at follow-up (months)	−.07	-				
3. Maternal pandemic-related stress	.02	.15*	-			
4. Prenatal maternal depression	−.04	−.03	.47**	-		
5. Postnatal maternal depression	−.08	.01	.33**	.59**	-	
6. Infant negative affect	−19**	.09	.17**	.01	.17**	-
Mean	8.97	13.37	0.00	9.29	7.62	4.35
*SD*	4.04	2.18	1.00	4.92	5.08	0.98
Range	0.4–14.6	0.7–17.1	−2.4 to 2.3	0–25	0–23	1.8–6.8

Note:

** *p* < .01

* *p* < .05.

**Table 3.** COVID-19-related stress: PCA component loadings

	Factor 1	Factor 2	Factor 3
Concern about concern about changes to medical birthing team	0.709	−0.103	−0.071
Concern about caring for the child after birth	0.650	−0.006	−0.101
Valance of the impact of the pandemic on daily life	−0.629	−0.172	0.025
Concern about social support during birth	0.629	−0.064	0.011
Impact of the pandemic on daily life	0.609	0.087	0.055
Concern about reduced access to resources	0.589	0.148	0.142
Distress about disrupted social support	0.536	−0.012	0.024
Concern about the child’s health	0.503	−0.040	0.181
Current financial impact	−0.069	0.858	0.004
Expected future financial impact	0.094	0.796	−0.016
Concern about own health	−0.045	0.025	0.901
Concern about family’s health	0.076	−0.039	0.861
Eigenvalue	3.68	1.31	1.21
Variance explained	30.66	10.95	10.09

**Table 4.** Path model estimates

Paths	*β*	*SE*	95% CI	*p*	*q*
*Outcome: Postnatal maternal depression*					
Prenatal maternal depression	0.57	0.05	0.47, 0.66	<.001	<.001
COVID-19 stress	0.06	0.06	−0.05, 0.16	.187	0.342
Income-to-needs ratio	−0.06	0.05	−0.15, 0.04	.260	0.409
*Outcome: infant negative affect*					
COVID-19 stress	0.16	0.06	0.05, 0.28	.006	0.013
Income-to-needs ratio	−0.19	0.06	−0.30, −0.08	.001	0.004
Child sex	0.003	0.07	−0.13, 0.13	.856	0.856
Child age at follow-up	0.05	0.06	−0.07, 0.17	.392	0.539
Covariances	*r*	*SE*	95% CI	*p*	*q*
Prenatal depression ↔ COVID-19 stress	0.47	0.05	0.38, 0.56	<.001	<.001
Prenatal depression ↔ Income-to-needs ratio	−0.04	0.06	−0.16, 0.08	.493	0.603
COVID-19 stress ↔ Income-to-needs ratio	0.02	0.06	−0.10, 0.14	.778	0.856
Postnatal maternal depression ↔ Infant negative affect	0.19	0.06	0.08, 0.31	.001	0.003

*Note. β* represents standardized beta coefficient; *r* represents correlation coefficient; *q* represents FDR corrected *p* value.
