# Supplementary material for: Maternal–prenatal stress and depression predict infant temperament during the COVID-19 pandemic
Source: Dev Psychopathol. Author manuscript; Available in PMC 2024 Aug 1. (PMC10164837; doi:10.1017/S0954579422001055)
Supplement: 1 [file NIHMS1834100-supplement-1.docx]

|  | Count | Percent |
| --- | --- | --- |
| Maternal age | 21 | 2.9 |
| Income-to-needs ratio | 1 | 0.1 |
| Education | 27 | 3.7 |
| Depressive symptoms | 8 | 0.8 |
| Concern about social support during birth | 4 | 0.6 |
| Concern about changes to medical birthing team | 2 | 0.3 |
| Concern about caring for the child after birth | 12 | 1.7 |

Supplemental material

Table S1. *Missing Data at Baseline Assessment*

Among the 725 pregnant people who completed all questionnaires at baseline, no data were missing more than 5.0% for relevant study variables: maternal age, income-to-needs ratio, education, race, depressive symptoms, concern about changes to medical birthing team, concern about caring for the child after birth, concern about social support during birth, valence of the impact of the pandemic on daily life, concern about reduced access to resources, distress about disrupted social support, concern about the child’s health, current financial impact, expected future financial impact, concern about own health, and concern about family’s health. Variables with any missing data are shown in Table S1. Little’s test was conducted and indicated that these data were missing completely at random (*X*^2^(11)=12.42, *p*=.333).

Table S2. *Missing Data at Follow-up Assessment*

|  | Count | Percent |
| --- | --- | --- |
| Maternal age | 6 | 2.0 |
| Education | 5 | 1.7 |
| Depressive symptoms (follow-up) | 5 | 1.7 |
| Concern about social support during birth | 1 | 0.3 |
| Concern about changes to medical birthing team | 1 | 0.3 |

Among the 296 pregnant people who completed all questionnaires at follow-up, no data were missing more than 5.0% for relevant study variables: maternal age, income-to-needs ratio, education, race, baseline and follow-up depressive symptoms, concern about changes to medical birthing team, concern about caring for the child after birth, concern about social support during birth, valence of the impact of the pandemic on daily life, concern about reduced access to resources, distress about disrupted social support, concern about the child’s health, current financial impact, expected future financial impact, concern about own health, concern about family’s health, and infant negative affect. Variables with any missing data are shown in Table S2. Little’s test was conducted and indicated that these data were missing completely at random (*X*^2^(18)=12.01, *p*=.894).

Figure S1a-e. *Scatterplots*


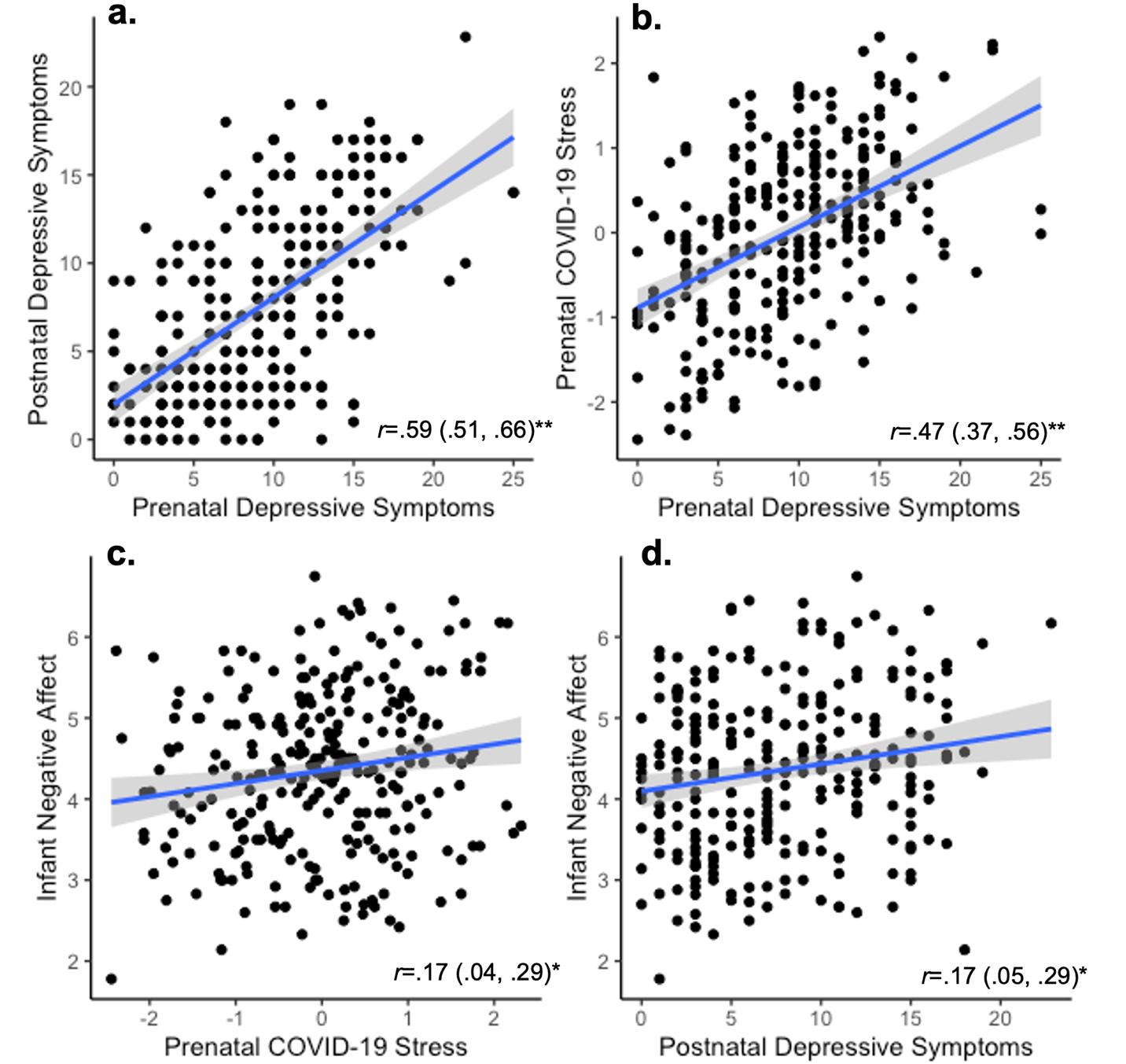


*Note*: *r*=Pearson’s correlation, numbers in parentheses are upper and lower bounds of 95% confidence intervals. ***p*<.001, **p*<.01.
